# Amino-Functionalization of Carbon Nanotubes by Using a Factorial Design: Human Cardiac Troponin T Immunosensing Application

**DOI:** 10.1155/2014/929786

**Published:** 2014-07-13

**Authors:** Tatianny A. Freitas, Alessandra B. Mattos, Bárbara V. M. Silva, Rosa F. Dutra

**Affiliations:** Biomedical Engineering Laboratory, Federal University of Pernambuco, Avenida Professor Moraes Rego 1235, 50670-901 Recife, PE, Brazil

## Abstract

A simple amino-functionalization method for carbon nanotubes and its application in an electrochemical immunosensor for detection of the human cardiac troponin T are described. Amino-functionalized carbon nanotubes allow oriented antibodies immobilization via their Fc regions, improving the performance of an immunosensor. Herein multiwalled carbon nanotubes were amino-functionalized by using the ethylenediamine reagent and assays were designed by fractional factorial study associated with Doehlert matrix. Structural modifications in the carbon nanotubes were confirmed by Fourier transform infrared spectroscopy. After amino-functionalization the carbon nanotubes were attached to screen-printed carbon electrode and a sandwich-type immunoassay was performed for measuring the cardiac troponin T. The electrochemical measurements were obtained through hydrogen peroxide reaction with peroxidase conjugated to the secondary antibody. Under optimal conditions, troponin T immunosensor was evaluated in serum samples, which showed a broad linear range (0.02 to 0.32 ng mL^−1^) and a low limit of detection, 0.016 ng mL^−1^. This amino platform can be properly used as clinical tool for cardiac troponin T detection in the acute myocardial infarction diagnosis.

## 1. Introduction

The screen-printing technology is a well-established and practical approach ideal for development of electrochemical point-of-care testing [1]. Screen-printed electrode (SPE) provides advantages such as easy miniaturization and portable instrumentation, making possible the on-site detection of different target analytes [2]. When compared with conventional electrodes, SPEs are inexpensive, being used as disposable due to their large-scale production capability. Several methods have been devoted to increase the surface area of the SPEs and enhance their sensitivity for electrochemical detection, including the application of nanomaterials [3–5]. Carbon nanotubes (CNTs) have attracted the interest of researchers in the field of electrochemical SPE immunosensors [6–8]. These nanomaterials specially combine several properties that improve the electrochemical performance, such as easy surface functionalization and increase in the amount of immobilized biomolecules and in the electron-transfer charge on the electrode surface [9].

The surface functionalization of the CNTs by linking specific functional groups has been a fundamental point for antibodies attaching as recognition element for immunosensing application. Carboxyl-terminated nanotubes have been also explored as essential functionalization strategy for covalent linkage of antibodies. However, this approach is limited because most antibodies contain amino groups randomly distributed, leading to multiple attachment sites. The random natures of this attachment sites can cause some loss of the antigen-binding activity due to the steric hindrance [10, 11]. The carboxylic groups present in Fc regions of the antibodies can be conveniently explored for oriented immobilization by exposing the Fab regions that exhibit a high affinity toward epitopes of the antigens. These groups can form stable amide bonds with the amino groups of the CNTs [12]. Thus, amino-functionalized nanotubes improve the reactivity of antigen-antibody recognition and the efficiency in immobilization process.

Techniques that are specifically intended for CNTs functionalization with amino groups comprise chemical treatments using acids, sheathing or wrapping of the CNTs with polymer chains [13, 14], grafting of CNTs with a thin layer of polymer chains based on plasma [15], or a combination of these [16]. However, these methods are generally a complicated process involving a long reaction time and several coupling reagents and require strictly controlled reaction conditions [17, 18]. In this work, a simple method based on fractional factorial design has been proposed for amino-functionalization of the CNTs using the ethylenediamine (EDA) as crosslink amino reagent. Under optimization process of functionalization, amino-CNTs were employed to develop an immunosensor for cardiac troponin T (cTnT), an important marker for acute myocardial infarction.

Cardiovascular diseases are the leading cause of death globally according to the World Health Organization statistics. Among the cardiovascular diseases, acute myocardial infarction (AMI) is one of the most serious diseases that extremely affect people's health [19]. In the past decades, cardiac troponins (cTnT and cTnI) have been recommended as the biomarkers of choice for the serological diagnosis and prognosis of AMI because of their high sensitivity and specificity [20–23]. In particular, the cTnT levels increase 2–4 h after the AMI symptoms and could be elevated up to 14 days after the acute episode of myocardial damage [24, 25]. Thus, the development of a rapid and practical immunosensor for the detection of the cTnT in serum samples from patients with myocardial infarction is desirable due to its roles in cardiospecific diagnosis, risk stratification, prognostic risk assessment, and therapeutic choices.

## 2. Materials and Methods

### 2.1. Reagents and Materials

Mouse monoclonal antibody against cTnT (mAb-cTnT), cTnT, and peroxidase conjugated mouse monoclonal antibody against cTnT (mAb-cTnT-HRP) were purchased from Calbiochem (Darmstadt, DEU). COOH-functionalized multiwalled carbon nanotubes (MWCNTs) were obtained from Dropsens (Oviedo, ESP). EDA,* N*-ethyl-*N*′-(3-dimethylaminopropyl) carbodiimide (EDC),* N*-hydroxysuccinimide (NHS), dimethylformamide (DMF), and glycine were acquired from Sigma-Aldrich (St. Louis, USA). Ethanol, sulfuric acid (H_2_SO_4_) (98%, w/v), and hydrogen peroxide (H_2_O_2_) (30%, w/v) were obtained from F. Maia (Cotia, BRA). All reagents were of analytical grade. The water used to prepare all solutions was obtained from a* Milli*-Q water purification system (Billerica, USA). All the solutions were freshly prepared prior to each experiment.

### 2.2. Serum Samples

The serum samples of cTnT were obtained from venous blood and immediately centrifuged for 120 s at 1150 rad/s. Aliquots of serum were quantified in an automatic system Roche Elecsys 2010 immunoassay analyzer based on electrochemiluminescence immunoassay (ECLIA) and the remaining serum, stored at −20°C, was used for the electrochemical measurements. The venous blood samples were collected from donor's patient from the Cardiac Emergency of Pernambuco (PROCAPE), the Hospital of Pernambuco State University, according to ethics committee's recommendations.

### 2.3. Apparatus and Measurements

Electrochemical studies were performed by using a *μ*Autolab III analysis system with GPES 4.9 software, Eco Chemie (Utrecht, NLD). Screen-printed carbon electrodes (SPCEs) were purchased from DropSens (Oviedo, ESP). These electrodes (reference 110) incorporate a conventional three-electrode configuration, printed on ceramic substrates (3.4 cm × 1.0 cm). Both working (disk-shaped 4.0 mm diameter) and counter electrodes were made of the carbon inks, whereas the pseudoreference electrode and electric contacts were made of silver.

Cyclic voltammetry (CV) was performed at 100 mV s^−1^ scan rate in presence of PBS (0.01 mol L^−1^, pH 7.0) used in all experiments as electrolyte support. The chronoamperometry was carried out in the same electrolyte at the potential of +0.3 V at 120 s. All experiments were executed at room temperature (24°C).

### 2.4. Characterization of the MWCNTs

The structural characterization of the MWCNTs was evaluated by Fourier transform infrared (FTIR) spectroscopy. The spectra were obtained by using a Bruker IFS 66 model FT-IR spectrometer in the region 4000 to 400 cm^−1^ by the standard KBr pellet technique. The modification of the SPE surface by deposition of the MWCNTs was characterized by scanning electron microscopy (SEM) using Philips XL30 FEG FE-SEM at 10 kV.

### 2.5. Amino-Functionalization of the MWCNTs

The carboxylic groups of the MWCNTs were activated by using a solution of 0.1 mol L^−1^ acetate buffer (pH 4.8) containing 0.1 mol L^−1^ EDC and 0.2 mol L^−1^ NHS [1 : 1]. At the same time, amino groups of the EDA were submitted to acid treatment in H_2_SO_4_ solution (0.1 mol L^−1^) for 2 h under stirring conditions at 60°C, changing their protonation state. After that, 1.0 mg activated MWCNTs was dispersed in 1.0 *μ*L EDA treated and it was stirred for 2.5 h at room temperature. The sample was centrifuged at 2000 ×g and washed five times with deionized water to remove acid residues. The resulting amino-MWCNTs were dried at 150°C and then dispersed in DMF under sonication during 2 h.

### 2.6. Electrode Preparation

Prior to use, the SPCE surface (0.125 cm^2^) was sonicated with ethanol and deionized water, respectively, for 2 min, to remove any organic contaminant. SPCE was coated with 3.0 *μ*L of the amino-MWCNTs on the working electrode surface and dried at 40°C for 5 min for absolute evaporation.

### 2.7. mAb-cTnT Immobilization

After being coated with amino-MWCNTs, the SPCE surface was incubated with an aliquot (3.0 *μ*L) of mAb-cTnT (1.0 *μ*g mL^−1^) for 1 h. In this concentration, the maximal current was measured as a result of antigen-antibody equilibrium demanded [26]. Subsequently, the electrode was rinsed by exhaustive PBS (pH 7.0) washing with the purpose of removing unbound mAb-cTnT. To avoid nonspecific binding, the modified electrode surface was blocked with 5.0 mmol L^−1^ glycine solution for 2 h.

### 2.8. Analytical Measurements

For evaluating the analytical response of the immunosensor, the modified electrodes were incubated with human serum samples spiked with different cTnT concentrations (0.02, 0.04, 0.08, 0.16, and 0.32 ng mL^−1^) for 30 min. After that, the electrode was incubated with mAb-cTnT-HRP for 1 h. The immunosensor response was evaluated by chronoamperometric assays at +0.3 V during 120 s. The amperometric detection of the cTnT was monitored through the electrocatalytic reduction reaction of the H_2_O_2_ by the HRP-labeled secondary mAb-cTnT. The measurements were performed in 0.1 mol L^−1^ PBS (pH 7.0), containing 1.5 mmol L^−1^ H_2_O_2_, at room temperature.

### 2.9. Multivariate Optimization

The screening of variables was accomplished using a 2^5–1^ fractional factorial design. Five variables were examined in two levels, lower (−) and upper (+). The subsequent factors and their levels were as follows: EDA concentration (50–100%), H_2_SO_4_ concentration (0.1–1.0 mol L^−1^), time of acid treatment of the EDA (1-2 h), amination time (2–4 h), and amino-MWCNT dispersion time (2–4 h) (Table 1). After establishing the variables, a Doehlert design was used for final optimization and then the response surface was obtained. These experiments were performed in a random order and the monitored parameter was the cathodic current established by CV. Data was processed using the STATISTICAL package program (version 6.0; Stat Soft, Inc., Tulsa, OK, USA).

## 3. Results and Discussion

### 3.1. Multivariate Optimization of the Amino-MWCNTs

Methods for chemical functionalization of MWCNTs have been reported by using different reactive groups, such as –NH_2_, –COOH, –OH, and –SH. Among these, the use of the –NH_2_ groups has been a viable alternative to attach antibodies to sensing surfaces in an oriented manner via amide bonds [27]. In this study, the amino-functionalization of the MWCNTs with EDA was investigated by using a fractional factorial design, which shows the influence of various factors on the experimental results and the optimum setting for each factor.

To optimize the different variables involving the amino-functionalization process of MWCNTs, a fractional factorial design associated with Doehlert matrix was used; EDA concentration (50%–100%), H_2_SO_4_ concentration (0.1–1.0 mol L^−1^), time of acid treatment of the EDA (1-2 h), amination time (2–4 h), and amino-MWCNT dispersion time (2–4 h) were established according to the results attained from the 2^5–1^ fractional factorial design and carried out in duplicate.

The Pareto diagram demonstrated that the most significant effects in the cathodic current were EDA concentration and time of acid treatment of the EDA. The positive values obtained in this study indicate that, by increasing these factors, the analytical signal will increase too. The factors were selected and simultaneously optimized by Doehlert design.

**Figure 1:**
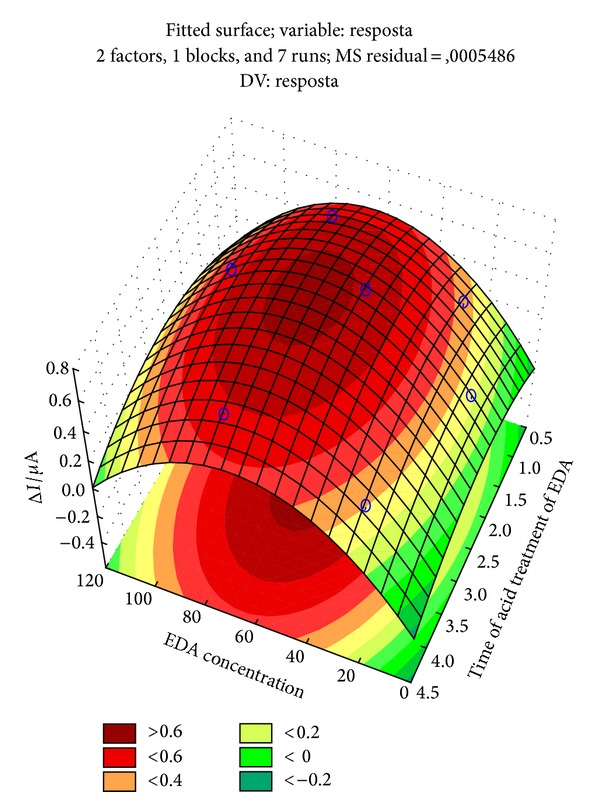
Surface response obtained from the Doehlert design employed for the optimization of the EDA concentration and time of acid treatment of the EDA in the amino-functionalization MWCNT procedure. The optimum conditions correspond to 70% and 2.5 h, respectively.

The EDA concentration was evaluated at five levels (10, 25, 50, 75, and 100%) and time of acid treatment of the EDA at three levels (1, 2, and 4 h).  Figure 1 shows the response surfaces obtained for the experiments considering the previously obtained effects. By analyzing the response surfaces, the optimum conditions of the experimental design generated the highest current peak. These conditions correspond to 70% and 2.5 h of the EDA concentration and time of acid treatment of the EDA, respectively.

### 3.2. Characterization of the MWCNTs

**Figure 2:**
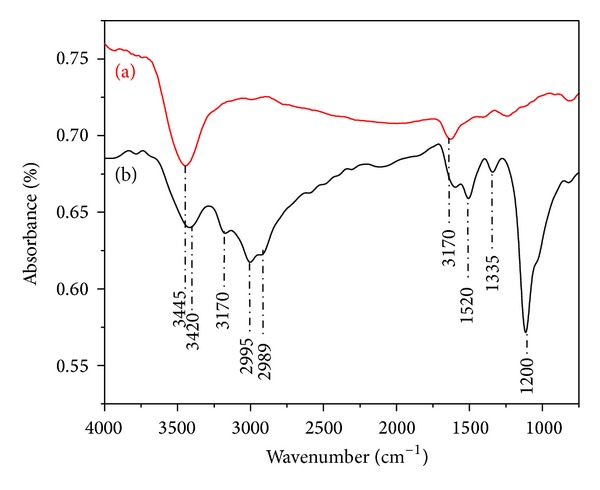
FTIR spectra of the MWCNTs (a) before and (b) after EDA treatment.

FTIR spectroscopy has proved to be a powerful tool to comply with the purpose of comprehensive characterization. The spectrum of the COOH-MWCNT in Figure 2(a) shows typical bands of the carboxylic groups at 3445 cm^−1^ corresponding to molecular stretching of –OH groups. The presence of the small peak at 1710 cm^−1^ is associated with the C=O stretching. These results were used as control of the amino-functionalization procedure. Figure 2(b) exhibits the spectra of the MWCNTs after EDA treatment. The peaks in the regions 3420 and 3170 cm^−1^ can be attributed to N–H stretch of the amino group. In addition, two peaks around 2995 cm^−1^ and 2989 cm^−1^ evidence the C−H stretching mode of the EDA molecule [28]. The appearance of new absorption bands at 1520 and 1340 cm^−1^ corresponds to N–H stretch of the amino group. The peak at 1120 cm^−1^ is ascribed to C–N stretching of amide groups [29].

The presence and location of the –NH_2_ and C–N bands in this spectrum provide strong evidence of the introduction of EDA moieties onto the MWCNT walls. Then, the obtained results showed a successful method for amino-functionalization of the MWCNTs.

**Figure 3:**
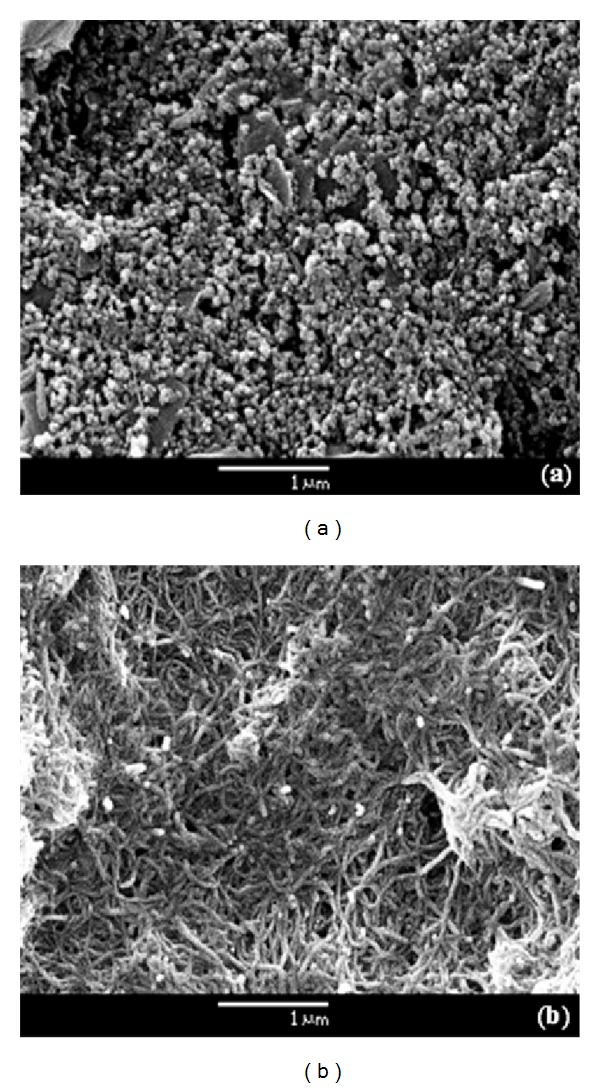
SEM image of working area of the SPCE (a) bare and (b) modified with amino-MWCNTs.

The SEM images were employed to morphologically characterize the modification of the electrode surface. The bare surface of the SPCE is showed in Figure 3(a). It was typically characterized by a great content of graphite particles covered with polymeric binder from the carbon ink [30]. The distribution of the graphite particles in the bare SPCE surface demonstrated a porous structure. The morphological changes in the sensing interface after treated amino-MWCNTs modification can been seen in Figure 3(b). This image reveals a homogeneous mesh of the amino-MWCNTs in the form of small bundles of tubes. The adsorbed amino-MWCNTs have resulted in a higher roughness as compared to unmodified SPCE, by favoring an increased active surface to further antibody immobilization. SEM analysis also showed that chemical modification of the MWCNTs in the amino-functionalization procedure did not affect the adsorption and formation of nanostructured film.

### 3.3. Immunosensor Preparation

**Figure 4:**
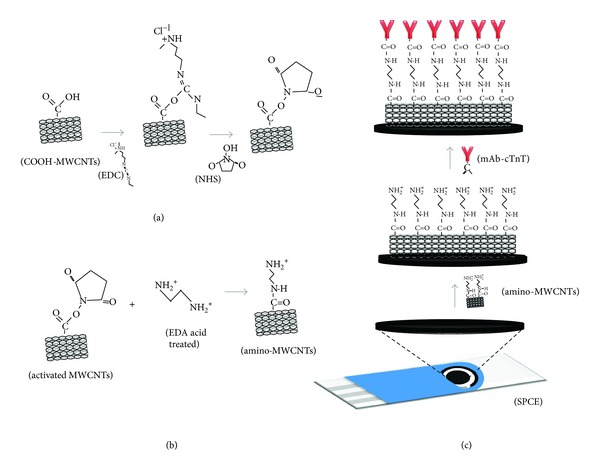
Schematic illustration of stepwise of the immunosensor: (a) activation of the MWCNTs, (b) amino-functionalization procedure, and (c) mAb-cTnT immobilization.

The scheme of stepwise immunosensor fabrication is illustrated in Figure 4. The activation of carboxylic acids of the MWCNTs with EDC/NHS chemical provides a reactive group for interaction with EDA (Figure 4(a)). The EDC/NHS enables the conversion of carboxylic groups to amino-reactive esters of the NHS [31]. These groups are susceptible to nucleophilic attack from amino group with charge excess of the EDA (Figure 4(b)). The reaction resulted in the formation of a stable covalent bond between the materials.

The use of the EDA to functionalize the MWCNTs with –NH_2_ groups showed a viable alternative to attach the –COOH groups present in Fc regions of the mAb-cTnT via amide bonds. The oriented immobilization via amide covalent binding influences the conformational stability and the activity of immobilized antibodies. Although more complex than other immobilization methods (i.e., physical adsorption), the attachment covalent provides a most-stable strategy for irreversible immobilization of the biomolecules [32, 33]. The advantage of low protein loss by desorption improves the stability and reproducibility of the device indicating that covalent immobilization is most desirable for several biosensor technologies [33]. The reactions of the mAb-cTnT immobilization procedure are demonstrated in Figure 4(c).

### 3.4. Optimization of the Experimental Conditions

**Figure 5:**
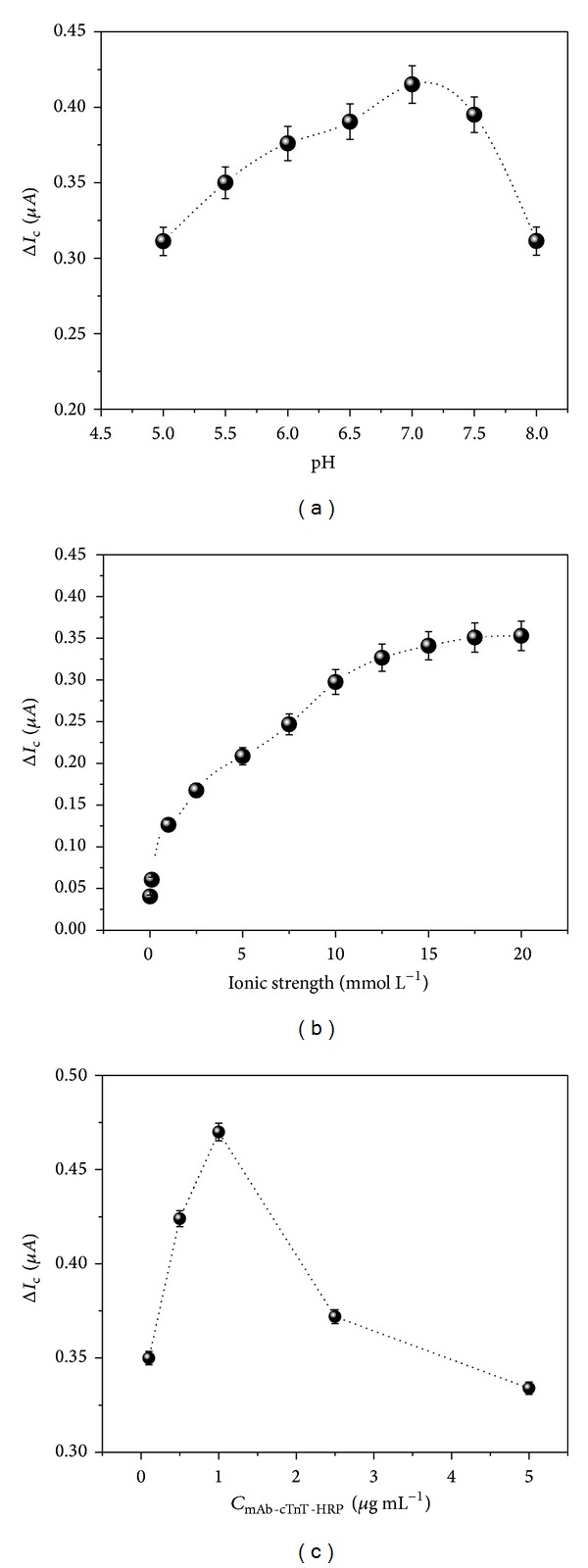
Influence of (a) pH values, (b) ionic strength of the PBS, and (c) the amount of the mAb-cTnT-HRP on the cathodic peak current of the SPCE. Measurements obtained by CVs experiments in presence of the 1.5 mmol L^−1^ H_2_O_2_.

Influence of the buffer pH is essential to analytical performance of the immunosensor, because the pH affects not only the electrochemical behavior of the sensor but also the bioactivity of the biomolecules [34]. The pH value of the 0.1 mol L^−1^ PBS was investigated in the range of 5.0 to 8.0. The measurements were obtained through CV assay in presence of the 1.5 mmol L^−1^ H_2_O_2_. The results in Figure 5(a) showed the effect of the pH on the reduction current of the SPCE. The cathodic current reached a maximum value at pH 7.0. Thus, the optimum pH was chosen for subsequent studies.

The ionic strength studies of the PBS were investigated. These experimental parameters can influence the charge transport of the electrolyte support in the electrochemical measurements of the SPCE [29]. For this assay, the SPCE was submitted to amperometric measurements in 1.5 mmol L^−1^ H_2_O_2_ solution diluted in different ionic strengths of PBS (0.01, 0.1, 1.0, 2.5, 5.0, 7.5, 10.0, 12.5, 15.0, 17.5, and 20.0 mmol L^−1^). The results exhibited a maximum reduction current at 0.01 mol L^−1^ of the PBS (Figure 5(b)).

In order to obtain an optimal response with the minimal amount of HRP-labeled antibody, the concentrations of the mAb-cTnT-HRP were varied. Herein, after the mAb-cTnT SPCE incubation with cTnT (2.5 ng  mL^−1^) for 1 h, the immunosensor was incubated at different concentrations of the mAb-cTnT-HRP (0.01 to 5.0 *μ*g mL^−1^). As can be seen in Figure 5(c), the reduction current increased with the increase of the anti-cTnT-HRP concentration up to 1.0 *μ*g mL^−1^.

### 3.5. Reproducibility and Stability

**Figure 6:**
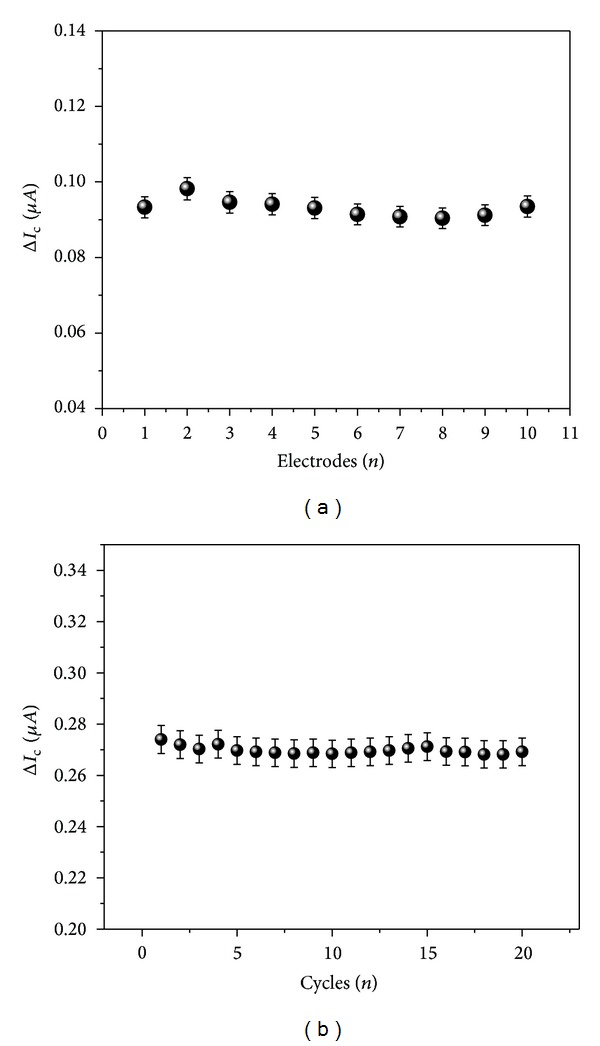
(a) Reproducibility and (b) repeatability of the SPCE to 2.5 ng mL^−1^ cTnT.

A key problem of immunosensors is their reproducibility and how long their devices can operate with a reproducible response [35]. In the reproducibility study, 10 different electrodes were prepared under the same conditions and evaluated with cTnT samples (2.5 ng mL^−1^). The immunosensor reached an acceptable reproducibility with a relative standard deviation (RSD) of 2.5% (Figure 6(a)). Regarding the repeatability, immunosensor has been shown to be significantly stable for 20 measurements in the same electrode registered in each 1 min interval. This statement was confirmed by low RSD (1.34%) (Figure 6(b)). These results can be attributed to strong interaction between the amino-MWCNT film and immobilized mAb-cTnT.

### 3.6. Determination of cTnT in Human Serum

**Figure 7:**
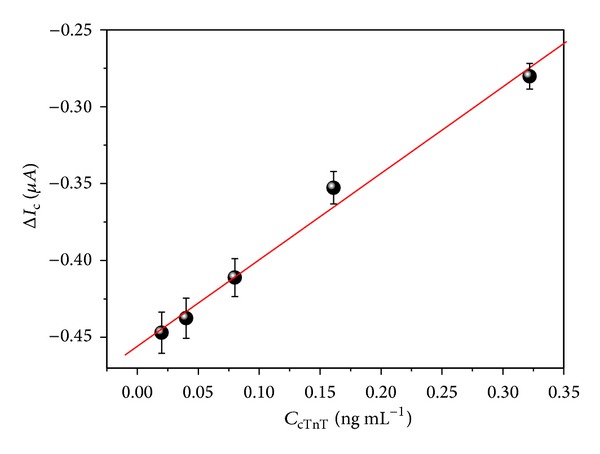
Calibration curve of the resulting immunosensor for cTnT detection in serum samples.

Human serum samples spiked with different cTnT concentrations were analyzed by chronoamperometric assays fixing the working potential at −0.3 V at 120 s (Figure 7). The amount of cTnT of serum samples was previously measured by Roche Elecsys 2010 immunoassay analyzer based on ECLIA. The immunosensor measurements showed a good agreement with the ECLIA method at 95% confident level when paired *t*-test was applied. As shown in Figure 7, the calibration plot exhibited a good linear correlation between 0.02 and 0.32 ng mL^−1^ cTnT (*r* = 0.985, *n* = 5, and *P* < 0.001). The limit of detection (LOD) of the SPCE was calculated according to the following equation:

(1) $$\begin{array}{c} \text{LOD}=\frac{3\text{SD}}{m}, \end{array}$$

where SD is standard deviation of the blank measurementand *m* is the slope of the linear part of the calibration curve [36]. The LOD was estimated in 0.016 ng mL^−1^ showing a high sensitivity; thereby this immunosensor can be useful for cTnT determination in clinical routine, since the cutoff is found at approximately 0.02 ng mL^−1^ in the AMI diagnosis. Since no mediators have been added in the electrochemical detection of the cTnT based on HRP reaction, the SPCE exhibited a LOD comparable with other cTnT immunosensors that make use of the mediators such as hydroquinone [26]. The properties of the MWCNTs at the sensor interface can dispense the employ of the mediators due to the ability of the nanomaterials to improve the electron-transfer reactions. The use of mediators can cause damage or passivation on the electrode surface, reducing its lifetime [37].

## 4. Conclusions 

A simpler method for amino-functionalization of the MWCNTs than the previously described was proposed. It does not require time-consuming and costly multistep reactions, several coupling reagents, or strictly controlled processes. The experimental conditions were optimized using a fractional factorial design showing that protonation time and EDA concentration are dependent parameters for successful MWCNTs functionalization. Oriented immobilization of anti-cTnT and development of an immunosensor for cTnT with high sensitivity and reproducibility were possible.

## Figures and Tables

**Table 1:** Experimental factors and their levels employed in 2^5−1^ fractional factorial design for amino-functionalization of the MWCNT procedure.

Experiment	EDA concentration − (50%) + (100%)	H_2_SO_4_ concentration − (0.1 mol L^−1^) + (1.0 mol L^−1^)	Time of acid treatment of the EDA − (1 h) + (2 h)	Amination time − (2 h) + (4 h)	Amino-MWCNT dispersion time − (2 h) + (4 h)	Δ*I* (*μ*A) mean values
1	−	−	−	−	+	0.5757
2	+	−	−	−	−	0.4328
3	−	+	−	−	−	0.2803
4	+	+	−	−	+	0.2803
5	−	−	+	−	−	0.4131
6	+	−	+	−	+	0.4278
7	−	+	+	−	+	0.6383
8	+	+	+	−	−	0.3049
9	−	−	−	+	−	0.4065
10	+	−	−	+	+	0.3443
11	−	+	−	+	+	0.4319
12	+	+	−	+	−	0.2983
13	−	−	+	+	+	0.2492
14	+	−	+	+	−	0.2131
15	−	+	+	+	−	0.4820
16	+	+	+	+	+	0.4475

## References

[1] Li M, Li YT, Li DW, Long YT (2012). Recent developments and applications of screen-printed electrodes in environmental assays—a review. *Analytica Chimica Acta*.

[2] Serrano N, Díaz-Cruz JM, Ariño C, Esteban M (2010). Ex situ deposited bismuth bilm on screen-printed carbon electrode: a disposable device for stripping voltammetry of heavy metal ions. *Electroanalysis*.

[3] Díaz -González M, Hernández-Santos D, González-García MB, Costa-García A (2005). Development an immunosensor the determination rabbit IgG using streptavidin modified screen-printed carbon electrodes. *Talanta*.

[4] Ekabutr P, Chailapakul O, Supaphol P (2013). Modification of disposable screen-printed carbon electrode surfaces with conductive electrospun nanofibers for biosensor applications. *Journal of Applied Polymer Science*.

[5] Justino CIL, Rocha-Santos TAP, Duarte AC (2013). Advances in point-of-care technologies with biosensors based on carbon nanotubes. *TrAC Trends in Analytical Chemistry*.

[6] Willner I, Willner B (2010). Biomolecule-based nanomaterials and nanostructures. *Nano Letters*.

[7] Jacobs CB, Peairs MJ, Venton BJ (2010). Review: carbon nanotube based electrochemical sensors for biomolecules. *Analytica Chimica Acta*.

[8] Vashist SK, Zheng D, Al-Rubeaan K, Luong JHT, Sheu F-S (2011). Advances in carbon nanotube based electrochemical sensors for bioanalytical applications. *Biotechnology Advances*.

[9] Singh C, Srivastava S, Ali MA (2013). Carboxylated multiwalled carbon nanotubes based biosensor for aflatoxin detection. *Sensors and Actuators B: Chemical*.

[10] Peluso P, Wilson DS, Do D (2003). Optimizing antibody immobilization strategies for the construction of protein microarrays. *Analytical Biochemistry*.

[11] Chen C, Baeumner A, Durst R (2005). Protein G-liposomal nanovesicles as universal reagents for immunoassays. *Talanta*.

[12] Wan Y, Su Y, Zhu X, Liu G, Fan C (2013). Development of electrochemical immunosensors towards point of care diagnostics. *Biosensors and Bioelectronics*.

[13] Chiu PW, Duesberg GS, Dettlaff-Weglikowska U, Roth S (2002). Interconnection of carbon nanotubes by chemical functionalization. *Applied Physics Letters*.

[14] Hill DE, Lin Y, Rao AM, Allard LF, Sun Y-P (2002). Functionalization of carbon nanotubes with polystyrene. *Macromolecules*.

[15] Shi D, Lian J, He P (2003). Plasma coating of carbon nanofibers for enhanced dispersion and interfacial bonding in polymer composites. *Applied Physics Letters*.

[16] Kim SW, Kim T, Kim YS (2012). Surface modifications for the effective dispersion of carbon nanotubes in solvents and polymers. *Carbon*.

[17] Castro MRS, Schmidt HK (2008). Preparation and characterization of low- and high-adherent transparent multi-walled carbon nanotube thin films. *Materials Chemistry and Physics*.

[18] Chiu W-M, Chang Y-A (2008). Chemical modification of multiwalled carbon nanotube with the liquid phase method. *Journal of Applied Polymer Science*.

[19] White HD, Chew DP (2008). Acute myocardial infarction. *The Lancet*.

[20] Mohammed AA, Januzzi JL (2010). Clinical applications of highly sensitive troponin assays. *Cardiology in Review*.

[21] Apple FS, Wu AHB, Jaffe AS (2002). European Society of Cardiology and American College of Cardiology guidelines for redefinition of myocardial infarction: How to use existing assays clinically and for clinical trials. *The American Heart Journal*.

[22] Zhang B, Morales A, Peterson R, Tang L, Ye JY (2014). Label-free detection of cardiac Troponin I with a photonic crystal biosensor. *Biosensors and Bioelectronics*.

[23] Guy MJ, Chen YC, Clinton L (2013). The impact of antibody selection on the detection of cardiac troponin I. *Clinica Chimica Acta*.

[24] Shen J, Huang W, Wu L, Hu Y, Ye M (2007). Thermo-physical properties of epoxy nanocomposites reinforced with amino-functionalized multi-walled carbon nanotubes. *Composites A: Applied Science and Manufacturing*.

[25] Yang Z, Min Zhou D (2006). Cardiac markers and their point-of-care testing for diagnosis of acute myocardial infarction. *Clinical Biochemistry*.

[26] Mattos AB, Freitas TA, Kubota LT, Dutra RF (2013). An o-aminobenzoic acid film-based immunoelectrode for detection of the cardiac troponin T in human serum. *Biochemical Engineering Journal*.

[27] Sham M-L, Kim J-K (2006). Surface functionalities of multi-wall carbon nanotubes after UV/Ozone and TETA treatments. *Carbon*.

[28] Xiong J, Zheng Z, Qin X, Li M, Li H, Wang X (2006). The thermal and mechanical properties of a polyurethane/multi-walled carbon nanotube composite. *Carbon*.

[29] Shen J, Huang W, Wu L, Hu Y, Ye M (2007). Thermo-physical properties of epoxy nanocomposites reinforced with amino-functionalized multi-walled carbon nanotubes. *Composites A: Apllied Science and Manufacturing*.

[30] Kadara RO, Jenkinson N, Banks CE (2009). Characterisation of commercially available electrochemical sensing platforms. *Sensors and Actuators B Chemical*.

[31] Pei Z, Anderson H, Myrskog A, Dunér G, Ingemarsson B, Aastrup T (2010). Optimizing immobilization on two-dimensional carboxyl surface: pH dependence of antibody orientation and antigen binding capacity. *Analytical Biochemistry*.

[32] Williams RA, Blanch HW (1994). Covalent immobilization of protein monolayers for biosensor applications. *Biosensors and Bioelectronics*.

[33] Makaraviciute A, Ramanaviciene A (2013). Site-directed antibody immobilization techniques for immunosensors. *Biosensors and Bioelectronics*.

[34] Ruiyi L, Qianfang X, Zaijun L, Xiulan S, Junkang L (2013). Electrochemical immunosensor for ultrasensitive detection of microcystin-LR based on graphenegold nanocomposite/functional conducting polymer/gold nanoparticle/ionic liquid composite film with electrodeposition. *Biosensors and Bioelectronics*.

[35] Ramírez NB, Salgado AM, Valdman B (2009). The evolution and developments of immunosensors for health and environmental monitoring: problems and perspectives. *Brazilian Journal of Chemical Engineering*.

[36] Long GL, Winefordner JD (1983). Limit of detection: a closer look at the IUPAC definition. *Analytical Chemistry*.

[37] Gomes-Filho SLR, Dias ACMS, Silva MMS, Silva BVM, Dutra RF (2013). A carbon nanotube-based electrochemical immunosensor for cardiac troponin T. *Microchemical Jounal*.

